# Correlation between mosquito larval density and their habitat physicochemical characteristics in Mazandaran Province, northern Iran

**DOI:** 10.1371/journal.pntd.0005835

**Published:** 2017-08-18

**Authors:** Seyed Hassan Nikookar, Mahmoud Fazeli-Dinan, Shahyad Azari-Hamidian, Seyed Nouraddin Mousavinasab, Mohsen Aarabi, Seyyed Payman Ziapour, Yahya Esfandyari, Ahmadali Enayati

**Affiliations:** 1 Student Research Committee, Department of Medical Entomology and Vector Control, School of Public Health and Health Sciences Research Center, Mazandaran University of Medical Sciences, Sari, Iran; 2 Assistant Professor, Department of Medical Entomology and Vector Control, School of Public Health and Health Sciences Research Center, Mazandaran University of Medical Science, Sari, Iran; 3 Associate Professor, School of Health and Research Center of Health and Environment, Guilan University of Medical Sciences, Rasht, Iran; 4 Associate Professor, Department of Biostatistics, School of Public Health, Mazandaran University of Medical Sciences, Sari, Iran; 5 Assistant Professor, Department of Epidemiology, Faculty of Medicine, Mazandaran University of Medical Sciences, Sari, Iran; 6 Department of Parasitology, North Research Center, Pasteur Institute of Iran, Amol, Iran; 7 Ph. D student, Department of Environmental Engineering, School of Public Health and Health Sciences Research Center, Mazandaran University of Medical Sciences, Sari, Iran; 8 Head of Medical Entomology Department, School of Public Health and Health Sciences Research Center, Mazandaran University of Medical Sciences, Sari, Iran; North Carolina State University, UNITED STATES

## Abstract

Characteristics of mosquito larval habitats are important in determining whether they can survive and successfully complete their developmental stages. Therefore, data on the ecological factors affecting mosquito density and abundance especially the physicochemical properties of water of their breeding sites, can possibly be helpful in implementing larval management programs. Mosquito larvae were collected using a standard 350 ml dipper from fixed habitats including: artificial pool, river edge, creek and etc, in 30 villages of 16 counties from May-December 2014. Water samples were collected during larval collection and temperature (°C), acidity (pH), turbidity (NTU), electrical conductivity (μS/cm), alkalinity (mg/l CaCO_3_), total hardness (mg/l), nitrate (mg/l), chloride (mg/l), phosphate (mg/l) and sulphate (mg/l) were measured using standard methods. Spearman correlation coefficient, Kruskal-Wallis test of nonparametric analysis, Chi-square (χ^2^) analysis, regression analysis and C_8_ interspecific correlation coefficient were used for data analysis. A total of 7,566 mosquito larvae belonging to 15 species representing three genera were collected from fixed larval breeding places. *Culex pipiens* was the dominant species except in four villages where *An*. *maculipennis* s.l. and *Cx*. *torrentium* were predominant. There was a significant positive correlation between the density of *Cx*. *pipiens* and electrical conductivity, alkalinity, total hardness and chloride, whereas no significant negative correlation was observed between physicochemical factors and larval density. The highest interspecific association of up to 0.596 was observed between *An*. *maculipennis* s.l/*An*. *pseudopictus* followed by up to 0.435 between *An*. *maculipennis* s.l*/An*. *hyrcanus* and *An*. *hyrcanus/An*. *pseudopictus*. The correlations observed between physicochemical factors and larval density, can possibly confirm the effect of these parameters on the breeding activities of mosquitoes, and may be indicative of the presence of certain mosquito fauna in a given region.

## Author summary

Determination of association between mosquito larval abundance and physicochemical factors in aquatic habitats in Mazandaran Province was the purpose of this study. In 30 villages of 16 counties in the province, *Culex pipiens*, *Cx*. *tritaeniorhynchus* and *An*. *maculipennis* s.l. were the most abundant species discovered in larval habitats. The pair of species *An*. *maculipennis* s.l/*An*. *pseudopictus* has the highest interspecific association in the area. *Culex pipiens* showed the greatest compatibility compared with other species with a range of densities from the lowest to the highest in different habitats containing varying levels of physicochemical factors. Measuring physicochemical factors of water in larval habitats indicated that values of chloride, electrical conductivity, alkalinity and total hardness were significantly associated with the larval abundance in different larval habitats. The electrical conductivity, alkalinity, total hardness and chloride had significant positive correlation with the density of *Cx*. *pipiens*, whereas no negative correlation was obtained between larval density and physicochemical factors. These differences in preferences and association with varying levels of physicochemical factors can have vector-borne diseases control implications, because habitat changes through the manipulation of these factors to the detriment of vector species can ultimately lead to reduced abundance of vector species. The data may also be useful to predict changes in the densities of mosquito larvae or their transition should natural or artificial environmental modifications lead to alteration in the physicochemical factors.

## Introduction

Water characteristics of breeding places are important for oviposition and development of mosquitoes [1]. Different characteristics of the oviposition sites such as: vegetation, temperature, turbidity, pH, concentration of ammonia, nitrite and nitrate, sulphate, phosphate, chloride, calcium, and water hardness affect mosquito larval density [2–4]. Changing these factors in larval habitats may create conditions favorable or unfavorable for mosquito biology [5]. Temperature lower than 14–16°C and higher than 30°C reduces the rate of larval development of many species [2,6]. Larvae of most mosquito species are found in nature in pH 3.3–10.5 [2]. Studies in several micro and macrohabitats have revealed that distribution of *Cx*. *pipiens*, *Cs*. *longiareolata*, *Cx*. *antennatus*, *Oc*. *caspius*, *Cx*. *vagans*, *Cx*. *decens*, *Cx*. *perexiguus*, *Cx*. *univittatus*, *An*. *multicolor* are directly correlated with temperature, ammonia, nitrate, pH, dissolved oxygen and salinity [7,8]; *An*. *culicifacies* with temperature and dissolved oxygen [1,9]; *Ae*. *albopictus* with conductivity, total dissolved solids, nitrate, phosphate, sulphate, turbidity and salinity [10,11]; and *An*. *varuna* with calcium [1]. While larval densities of *Cx*. *pipiens*, *Cx*. *perexiguus* and *Ae*. *albopictus* are not directly affected by total nitrogen content, salinity and turbidity [8,10,11]. Dissolved nitrogen content can be a limiting factor on larval growth of the genus *Aedes* (formerly *Ochlerotatus*) by indirect effects on the trophic structure of tree-hole environments [12]. Although abundance of larvae can be associated with the soluble nitrate and phosphate levels in an area [13], it can equally be independent of those in another area because the nitrogen or phosphorus in water bodies can result in eutrophication and oxygen depletion, harmful algal blooms, toxic effects on fish and some aquatic organisms and overall reductions in aquatic biodiversity [4,14]. Therefore other factors can be effective in predicting larval density in different areas [15]. For example, the vegetation of mosquito larval habitats is considered as an important factor in the process of egg-laying and density of mosquito larvae [16]. Tall emergent aquatic plants can cover the surface and decrease mosquito larval density by acting as a barrier for egg-laying female. They may cause microbial growth and produce a high variety of predators [17].

Not much data currently exists regarding the physiochemical characteristics of mosquito larval habitats in Iran. Most of the available data is on anopheline mosquito larval habitats. The distribution of *An*. *sacharovi* is significantly associated with calcium bicarbonate, sodium sulphate and salinity in larval habitats in Ardebil Province, northwestern Iran[18]. Larval density of *An*. *culicifacies* s.l., *An*. *dthali*, *An*. *stephensi*, *An*. *superpictus*, *An*. *fluviatilis* s.l., *An*. *turkhudi*, *An*. *moghulensis*, and *An*. *apoci* is associated with temperature, EC, alkalinity, chloride and sulphate, total hardness, and dry residues in Bashagard and Rudan district, southern Iran [15,19]. A significant difference was observed between the density of *An*. *culicifacies* and phosphate, calcium and EC; *An*. *turkhudi* and pH, total hardness and nitrate; *An*. *superpictus* and total hardness and nitrate; *An*. *stephensi* and nitrate, *An*. *multicolor* and pH and sulphate in Iranshahr, southwestern Iran [20]. Temperature, pH, turbidity, EC, TDS, alkalinity, total hardness, calcium, chloride, fluoride, nitrite, nitrate, phosphate, sulphate were not significantly correlated with *An*. *claviger*, *An*. *marteri*, *An*. *superpictus*, *An*. *turkhudi*, *Cx*. *arbieeni*, *Cx*. *hortensis*, *Cx*. *mimeticus*, *Cx*. *modestus*, *Cx*. *pipiens*, *Cx*. *territans*, *Cx*. *theileri*, *Cs*. *longiareolata*, *Cs*. *subochrea*, *Oc*. *caspius* s.l. in Qom Province, central Iran [21]

To date, there is no information on physicochemical characteristics of mosquito larval habitats and coefficient of interspecific association (C_8_) in Mazandaran Province and this is the first such study in the province. Differences in environmental and geographical characteristics of the North of Iran in comparison with other provinces of the country have caused a fertile environment for the development of mosquitoes. The area had a history of diseases transmitted by vectors, the most important of which has been Malaria. Mazandaran has a unique environment fit for migratory birds and thousands of different wild bird species that spend winter in numerous fresh water lakes and wetlands across the province. West Nile virus is circulating in the province between the migratory birds and humans mostly by *Culex* mosquitoes [22,23]. Also, the environment may allow the invasion and establishment of *Aedes* vectors of dengue and Zika based on the risk assessment of the latter, a huge national research activity is underway to verify the risk. Information on the ecological factors affecting mosquito larval biology such as the physicochemical properties of the water of the breeding places and interspecific associations are important in survival, spatio-temporal distribution [24], biodiversity, affinity and association indices of disease vectors [25–27]. The information may serve as the basis for designing and implementation of adequate vector control programs [28]. Despite the voluminous literature on the distribution of mosquito larvae and physicochemical factors, the data set seems to be inconclusive in leading to a prediction of the presence of larvae in different habitats. Therefore more studies and systematic reviews with proper generalization are required. The present study was conducted to document the relationship between physicochemical characteristics of mosquito larval habitats and the presence or absence of a given species, density or diversity and interspecific associations of larvae in different habitats in Mazandaran Province.

## Methods

### Ethics statement

This research has been approved by the Ethic committee of Mazandaran University of Medical Sciences under the code 1017.

### Study area

**Fig 1:**
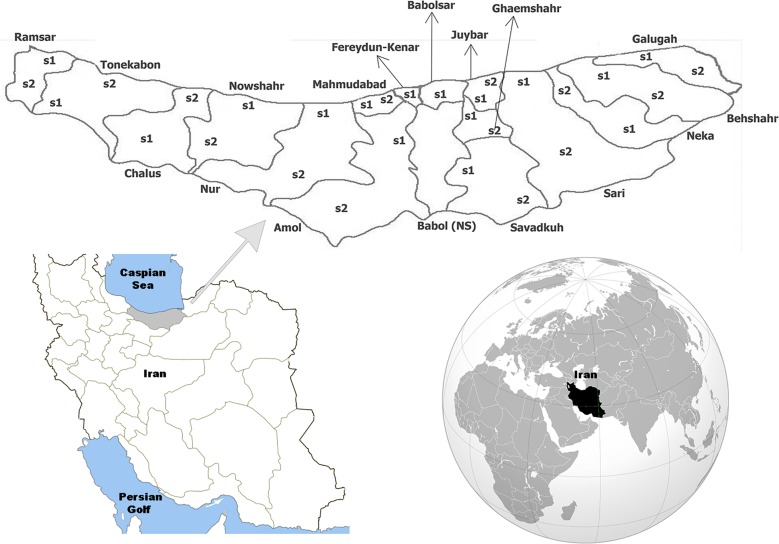
Map shows sampling places in various counties and villages of Mazandaran Province (s1 and s2 stand for station 1 and station 2). These comprise: Galugah County (s1: Nezammahale, s2: Tileno villages), Behshahr (s1: Hossein Abad, s2: Al Tappeh), Neka (s1: Chalmardi, s2: Komishan), Sari (s1: Qajar Kheil, s2: Dallak Kheil), Ghaemshahr (s1: Rostam kola, s2: Shahrud Kola), Savadkooh (s1: Sorkh Kola, s2: Andar Koli), Juybar (s1: Astanesar, s2: Pain Zarrin Kola), Babolsar (s1: Kikha Mahalleh), Fereydunkenar (s1: Firuzabad), Amol (s1: Qadi Mahalleh, s2: Razakeh), Mahmudabad (s1: Galesh Pol, s2: Bishe Kola), Noor (s1: Abbasa, s2: Karat Koti), Noshahr (s1: Aliabad Mir, s2: Shofeskaj), Chalos (s1: Sinava, s2: Zavat), Tonekabon (s1: Asadabad, s2: Soleymanabad) and Ramsar (s1: Shah Mansur Mahale, s2: Potak). Babol (NS: no sampling).

The study was carried out from May to December 2014 in Mazandaran Province located within the latitude of 35° 47′- 36° 35′ N and longitude of 50° 34′–54° 10′ E (Fig 1). The province has an area of 23756.4 km^2^ and a population of 3,073,943 (2011 census) [29]. It is located between Golestan Province in the East, Gilan Province in the West, Caspian Sea in the North and Tehran and Semnan Provinces in the South. The inhabitants are mainly involved in agriculture, animal husbandry, production of farmed fish and tourism industry. The climate varies from mild and humid in the Caspian Sea shore to moderate and cold in mountainous regions. The minimum and maximum mean annual temperatures and rainfall ranges between 1.2–29.2°C and 0–755.6 mm, respectively.

### Mosquito collection and identification

**Fig 2:**
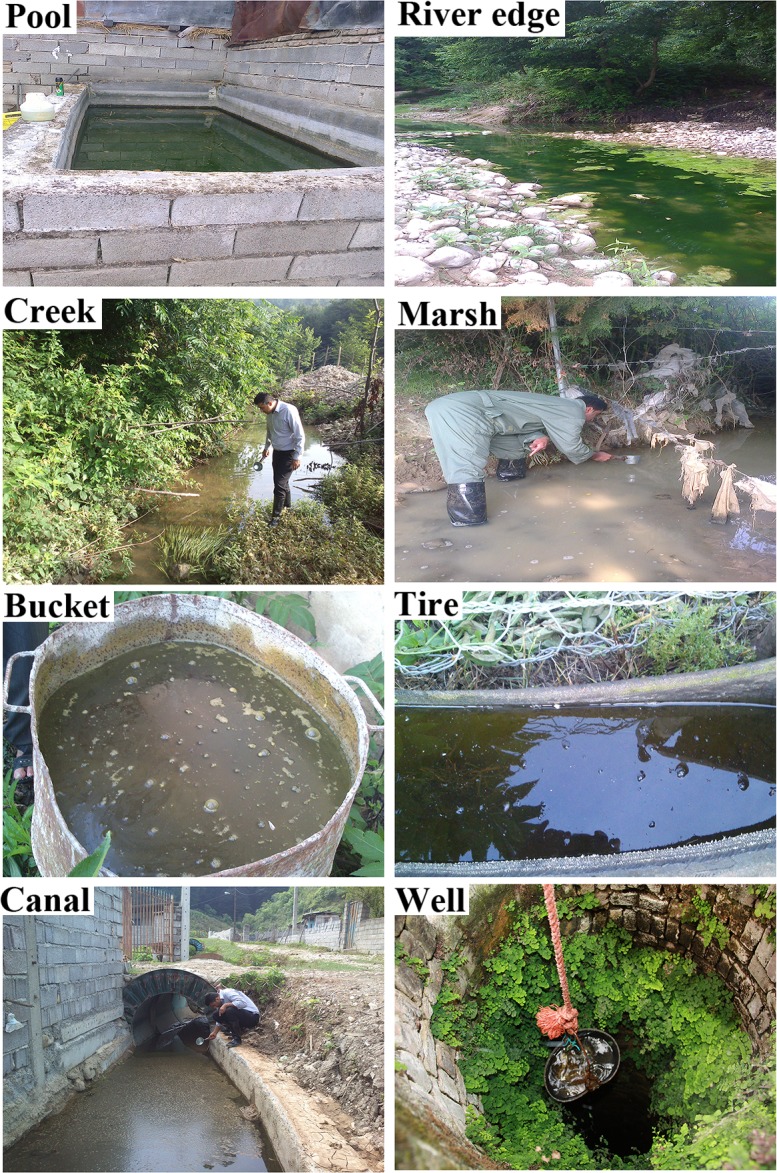
A variety of fixed habitats of mosquito larvae in Mazandaran Province, 2014 (Original photo).

Mosquito larvae were collected by standard 350 ml dipper from fixed oviposition sites (breeding places with permanent water during the sampling period) such as artificial pools, river edge, creek, marsh, large metal bucket, abandoned wells, water canals, pit with plastic floor (Fig 2), in 30 villages of 16 counties across the province (Fig 1). In each village, one fixed station was selected and visited for larval collection once a month. It is important to mention that one hundred staff members of the Mazandaran health centers were recruited and trained during two theoretical and practical workshops before undertaking sample collection. We met with county and village councils to seek cooperation of the villagers for assistance with sampling team. Collected larvae were conserved in lactophenol and were transferred to the Medical Entomology Laboratory at Faculty of Health, Mazandaran University of Medical Sciences. In the laboratory, microscope slides were prepared from each of the specimens using Berlese’s medium and larvae were identified by morphological characteristics according to appropriate keys [30,31].

### Physicochemical analyses

Water samples were collected from the same 30 fixed larval habitats in which dip samples had been collected in designated villages and were kept in one liter appropriately labeled polyethylene bottles. The bottles were placed inside an ice box and were sent for analysis to the Faculty of Health. Temperature (°C), acidity (pH), turbidity (NTU), electrical conductivity (EC) (Micro-Simens/cm) (μS/cm), alkalinity (mg/l CaCO_3_), total hardness (mg/l), nitrate (mg/l), chloride (mg/l), phosphate (mg/l) and sulphate (mg/l) of the water were measured. Water temperature and pH were measured on site using a thermometer and pH probe (Eutech-Cyberscan PH5500), turbidity using a turbidimeter device (2100P Portable Turbidimeter at Hach), EC using a conductometer device (EC LYTIC-AQUA (CON200) before dipping. Alkalinity, total hardness and chloride were determined using direct titration techniques and nitrate, sulphate and phosphate were measured using a spectrophotometer device (Perkinelmer UV/Visible Lambda EZ201 and HACH). All analyses were conducted according to the standard methods used in Rice *et al*. [32].

### Statistical analysis

The species data from each site was summed up and collectively reported in a graph. The means and standard deviations of physicochemical parameters of each breeding site was calculated using SPSS software version 19. The presumption for normality was tested using the Shapiro-Wilk test. The physicochemical parameters among sites were compared by Kruskal-Wallis test of nonparametric analysis. Chi-square (χ^2^) analysis was performed to determine whether there was any significant difference in distribution of the species in different counties. The Spearman correlation coefficient was used to examine the relation of the mosquito larval densities to the physicochemical factors adjusted by types of habitats. Regression analysis between larval densities and physicochemical factor were also performed to clarify the relationship and r-squared values were calculated.

### Interspecific association

Hurlbert’s coefficient of interspecific association (C_8_) was used to measure the associations between co-occurring species using presence-absence data. Values of C_8_ range from -1 to +1 for negative and positive associations, respectively. Positive associations between species can probably show a common habitat preference or interspecific attraction, whereas negative associations may reveal different habitat preferences or interspecific repulsion. The formula used for calculating C_8_ is as follows:

$$C_{8}=\frac{ad-bc}{\left|ad-bc\right|}\left|\sqrt{\frac{{Obs}_{x^{2}}-{Min}_{x^{2}}}{{Max}_{x^{2}}-{Min}_{x^{2}}}}\right|$$

where a, b, c, and d are the values in four cells of a 2 × 2 contingency table; Obs χ^2^ is referred to the value of χ^2^ associated with the observed values of a, b, c and d; Max χ^2^ is referred to the value of χ^2^ when a is as large (if ad ≥bc) or as small (if ad < bc) as the marginal totals of the 2 × 2 table permit; Min χ^2^ is referred to the value of χ^2^ when the observed *a* differs from its expected value (â) by less than 1.00 [33].

## Results

**Fig 3:**
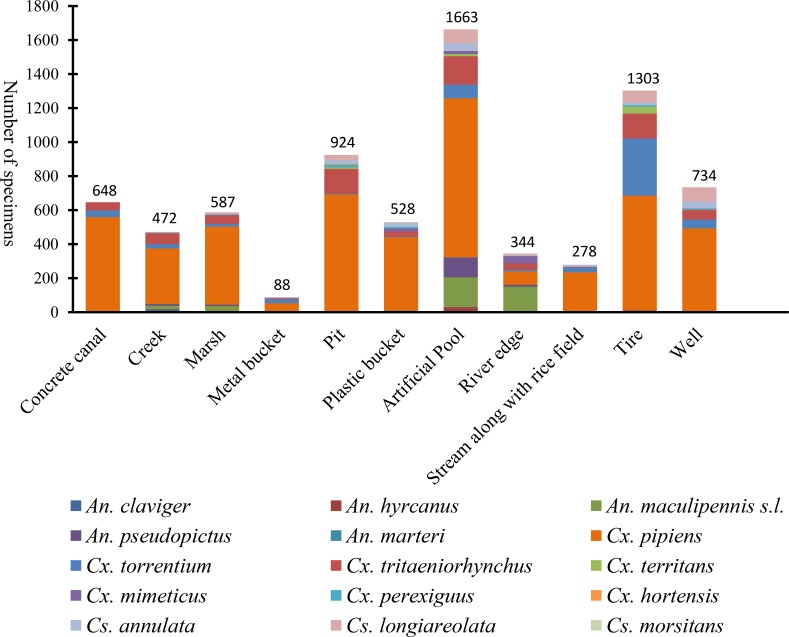
Composition of the mosquito larvae collected from eleven types of fixed larval habitats in Mazandaran Province, May- December 2014.

A total of 7,566 mosquito larvae from three genera and 15 species were collected from eleven different types of fixed oviposition sites. Using random effect model, it is observed that habitats type affect the abundance of species (P < 0.05). *Culex pipiens* (56.22%), *Cx*. *tritaeniorhynchus* (10.05%) and *An*. *maculipennis* s.l. (10.50%), were the most abundant species observed in artificial pool. whereas, *An*. *marteri* (0.06%), *Cx*. *hortensis* (0.15%) and *Cs*. *morsitans* (0.08%) were relatively uncommon in habitats such as concrete canal, artificial pool and tire; respectively (Fig 3).

Using the coefficient of interspecific association (C_8_), significant positive associations was determined between *An*. *maculipennis* s.l*/An*. *hyrcanus* and *An*. *hyrcanus/An*. *pseudopictus* of up to 0.435. This coefficient was up to 0.596 between *An*. *maculipennis* s.l/*An*. *pseudopictus* (Table 1).

**Table 1:** Coefficients of interspecific association (C_8_) for larvae of different species in eleven types of fixed habitats in Mazandaran Province, May-December 2014.

Species	*An*. *Claviger*	*An*. *hyrcanus*	*An*. *maculipennis* s.l	*An*. *pseudopictus*	*An*. *marteri*	*Cx*. *pipiens*	*Cx*. *torrentium*	*Cx*. *tritaeniorhynchus*	*Cx*. *territans*		*Cx*. *mimeticus*	*Cx*. *perexiguus*	*Cx*. *hortensis*	*Cs*. *annulata*	*Cs*. *longiareolata*	*Cs*. *morsitans*
*An*. *Claviger*	*	0	0	0	0	0	0	0	0	0		0	0	0	0	0
*An*. *hyrcanus*		*	0.435	0.435	0	0	0	0	0	0		0	0	0	0	0
*An*. *maculipennis* s.l			*	**0.596**	0	0	0	0	0	0		0	0	0	0	0
*An*. *pseudopictus*				*	0	0	0	0	0	0		0	0	0	0	0
*An*. *marteri*					*	0	0	0	0	0		0	0	0	0	0
*Cx*. *pipiens*						*	0	0	0	0		0	0	0	0	0
*Cx*. *torrentium*							*	0	0	0		0	0	0	0	0
*Cx*. *tritaeniorhynchus*								*	0	0		0	0	0	0	0
*Cx*. *territans*									*	0		0	0	0	0	0
*Cx*. *mimeticus*										*		0	0	0	0	0
*Cx*. *perexiguus*												*	0	0	0	0
*Cx*. *hortensis*													*	0	0	0
*Cs*. *annulata*														*	0	0
*Cs*. *longiareolata*															*	0
*Cs*. *morsitans*																*

There are significant differences in the distribution of the species in spatial scale (P < 0.05). *Culex pipiens* had the largest distribution and was the dominant species in all villages except Tileno, Zavat, Asad Abad and Shah Mansur Mahale. The highest and the lowest number and percentage of *Cx*. *pipiens* were recorded in the villages of Firozabad (558, 86.11%) and Tileno (3, 3.26%), respectively.

**Fig 4:**
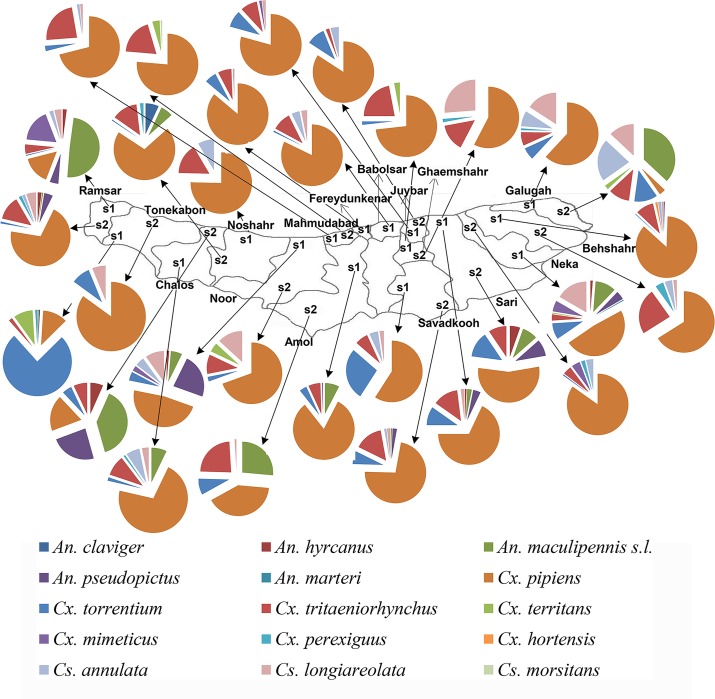
Distribution and number of mosquito larvae collected from fixed habitats in villages of Mazandaran Province, May- December 2014.

*Culex tritaeniorhynchus* was dispersed in all villages except Abbasa and Soleymanabad and showed the highest abundance in the village of Al Tappeh (95, 24.05%). *Anopheles maculipennis* s.l. were the dominant anopheline in the villages of Tileno, Zavat and Shah Mansur Mahale and showed the highest dispersion rate compared to the rest of the *Anopheles*. *Culiseta morsitans* and *An*. *marteri* showed the lowest distribution in the villages of Bishekola (0.76%) and Chalmardi (0.40%). Data regarding distribution of other species in spatial scale are shown in Fig 4.

Means and standard deviations of the physicochemical factors from larval habitats in different villages were calculated for each species. *Culiseta morsitans* was found in breeding sites with higher pH up to 7.44. It prefers lower temperature, alkalinity, nitrate, chloride and phosphate. *Anopheles marteri* prefers higher turbidity, alkalinity, total hardness, phosphate and sulphate, while *An*. *claviger*, *Cx*. *mimeticus* and *Cx*. *perexiguus* breed in habitats with higher temperature, EC and chloride, respectively. *Culex mimeticus*, *Cx*. *hortensis*, *Cx*. *torrentium* and *An*. *claviger* were collected in habitats with the lowest ranges of pH (6.88±0.40), turbidity (12.50±0.70), EC (69.42±1051.15), total hardness (192) and sulphate (14.18), respectively (Table 2). Ranges of the physicochemical parameters of larval habitats of different species are presented in Table 2.

**Table 2:** Means and standard deviations of physicochemical characteristics, along with occurrence of mosquito species in different larval habitats in Mazandaran Province, May-December 2014.

Occurrence in villages	Temperature (°C)	Acidity (pH)	Turbidity (NTU)	Electrical conductivity (μS/cm)	Alkalinity (mg/l CaCO_3_)	Nitrate (mg/l)	Total hardness (mg/l)	Chloride (mg/l)	Phosphate (mg/l)	Sulphate (mg/l)
*An*. *claviger* (1)	18	7.35	21	496	204	0.840	192	85.97	0.558	14.18
*An*. *hyrcanus* (7)	17.29± 0.95	7.22±0.35	42.57±62.72	705.71±832.65	245.14±179.31	2±2.24	372.57±331.11	134.10±267.26	0.99±1.22	117.77±156.58
*An*. *maculipennis* s.l. (14)	17.29±1.13	7.24±0.33	32.64±46.27	613.21±614.68	222.29±140.30	1.96±2.49	502.00±614.68	94.04±187.77	0.67±0.94	102.93±130.81
*An*. *pseudopictus* (10)	17.30±1.05	7.25±0.34	33.30±53.41	712.20±766.61	244.90±167.73	1.87±1.87	336.80±288.27	144.15±251.81	0.86±1.05	101.23±135.34
*An*. *marteri*(1)	16.00±0	7.6±0	97.00±0	1109.00±0	500.00±0	5.23±0	716.00±0	102.96±0	3.20±0	446.18±0
*Cx*. *pipiens*(30)	16.33±2.35	7.13±0.42	34.97±65.15	850.10±994.24	239.63±149.66	2.10±2.92	450.80±466.48	353.82±942.00	0.54±0.82	101.87±96.92
*Cx*. *torrentium*(26)	16.50±2.12	7.12±0.45	38.23±69.42	69.42±1051.15	248.81±158.39	2.00±2.85	479.23±494.97	211.54±366.40	0.52±0.76	102.62±101.02
*Cx*. *tritaeniorhynchus*(28)	16.25±2.41	7.14±0.44	36.96±67.06	885.43±1020.51	244.46±152.06	1.87±2.53	465.57±478.88	478.88±971.88	0.47±0.78	105.71±99.27
*Cx*. *territans*(6)	14.67±2.94	6.97±0.50	81.17±126.42	614.00±446.94	265.33±144.43	2.33±2.99	440±332.19	58.14±36.91	0.85±1.24	188.84±156.99
*Cx*. *mimeticus*(7)	16.57±0.97	6.88±0.40	29.71±33.97	1430.86±1395.97	336±227.69	3.26±2.97	588±386.43	332.03±517.66	1.15±1.21	155.50±142.36
*Cx*. *perexiguus*(8)	16.88±1.35	7.11±0.17	19±12.87	748.88±1139.53	239.50±216.74	2.38±2.75	612.50±812.35	826.24±1751.24	0.43±0.79	122.64±102.50
*Cx*. *hortensis* (2)	17	7.00±0.78	12.50±0.70	1169.50±590.43	368.50±96.87	1.37±0.27	440±39.59	279.41±273.56	0.58±0.10	146.13±4.94
*Cs*. *annulata* (15)	16.27±2.40	7.17±0.37	16.20±14.42	899.80±1116.41	253.67±174.98	1.24±1.45	537.60±607.43	572.28±1297.94	0.57±0.84	77.36±79.12
*Cs*. *longiareolata* (20)	16.05±2.06	7.13±0.46	35.95±73.21	761.55±824.98	231.25±128.02	2.02±2.95	474.40±514.07	388.47±1105.26	0.63±0.93	113.16±108.03
*Cs*. *morsitans* (1)	10	7.44	21	419	168	0.30	300	45.98	0	164.54

**Fig 5:**
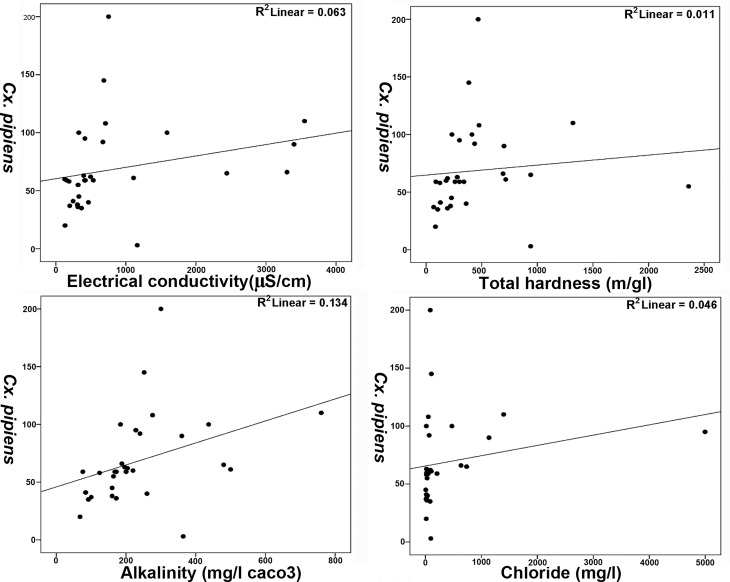
Regression relationships between *Cx*. *pipiens* and EC, alkalinity, total hardness and chloride of larval habitats in Mazandaran Province, August 2014.

Positive correlation exists between the larval abundance of *Cx*. *pipiens* and the physicochemical characteristics including EC, alkalinity, total hardness and chloride with Spearman rank correlations of 0.575 (P<0.001), 0.617 (P<0.001), 0.495 (P<0.005) and 0.539 (P<0.002), respectively. However, there was no significant negative correlation between physicochemical characteristics and larval abundance (Table 3). The regression relationship (R^2^) between *Cx*. *pipiens* and EC, alkalinity, total hardness and chloride were 0.11, 0.20, 0.01 and 0.06, respectively which is shown by Scatter plot (Fig 5).

**Table 3:** Correlation coefficient between larval density and physicochemical properties of larval habitats adjusted by the habitats types in Mazandaran Province, August 2014.

Species		Temperature (°C)	Acidity (pH)	Turbidity (NTU)	Electrical conductivity (μS/cm)	Alkalinity (mg/l CaCO_3_)	Nitrate (mg/l)	Total hardness (mg/l)	Chloride (mg/l)	Phosphate (mg/l)	Sulphate (mg/l)
*An*. *claviger*	Correlation Coefficient	0.174	0.118	0.107	0.054	0.032	0.011	-0.150	0.086	0.141	-0.268
	Sig.	0.357	0.534	0.572	0.778	0.866	0.955	0.428	0.652	0.456	0.152
*An*. *hyrcanus*	Correlation Coefficient	0.240	0.147	0.011	-0.184	-0.110	0.031	-0.150	-0.263	0.186	-0.054
	Sig.	0.201	0.440	0.955	0.330	0.562	0.871	0.428	0.160	0.325	0.776
*An*. *maculipennis* s.l.	Correlation Coefficient	0.346	0.241	0.113	-0.126	-0.099	0.111	-0.079	-0.241	0.146	-0.095
	Sig.	0.061	0.200	0.553	0.507	0.604	0.561	0.677	0.199	0.441	0.618
*An*. *pseudopictus*	Correlation Coefficient	0.274	0.226	-0.023	-0.152	-0.042	0.093	-0.168	-0.298	0.245	-0.115
	Sig.	0.142	0.229	0.904	0.422	0.825	0.627	0.376	0.109	0.192	0.544
*An*. *marteri*	Correlation Coefficient	-0.098	0.268	0.269	0.182	0.290	0.226	0.225	0.140	0.315	0.311
	Sig.	0.606	0.152	0.151	0.335	0.120	0.231	0.231	0.462	0.090	0.094
*Cx*. *pipiens*	Correlation Coefficient	-0.110	-0.271	0.044	**0.575**	**0.617**	0.077	**0.495**	**0.539**	-0.024	0.321
	Sig.	0.562	0.147	0.819	**0.001**	**<0.001**	0.684	**0.005**	**0.002**	0.901	0.084
*Cx*. *torrentium*	Correlation Coefficient	0.099	0.040	0.113	0.194	0.145	-0.139	0.146	-0.122	-0.075	0.174
	Sig.	0.602	0.834	0.552	0.305	0.444	0.463	0.440	0.520	0.692	0.358
*Cx*. *tritaeniorhynchus*	Correlation Coefficient	-0.219	-0.131	-0.240	0.287	0.227	0.130	0.136	0.320	-0.322	0.203
	Sig.	0.245	0.490	0.201	0.124	0.228	0.492	0.475	0.085	0.082	0.282
*Cx*. *territans*	Correlation Coefficient	-0.316	-0.312	0.092	-0.130	-0.001	-0.048	-0.085	-0.150	-0.017	0.134
	Sig.	0.088	0.094	0.629	0.495	0.996	0.802	0.655	0.428	0.930	0.479
*Cx*. *mimeticus*	Correlation Coefficient	-0.072	-0.201	0.044	0.262	0.232	0.220	0.254	0.136	0.277	0.221
	Sig.	0.706	0.287	0.816	0.162	0.218	0.243	0.175	0.475	0.139	0.240
*Cx*. *perexiguus*	Correlation Coefficient	-0.019	-0.132	0.044	-0.047	-0.020	0.312	-0.165	0.059	0.306	-0.315
	Sig.	0.920	0.486	0.818	0.806	0.916	0.094	0.382	0.755	0.100	0.090
*Cx*. *hortensis*	Correlation Coefficient	0.033	0.247	-0.032	0.225	0.247	0.075	0.097	0.204	0.098	0.139
	Sig.	0.864	0.188	0.866	0.231	0.189	0.693	0.612	0.280	0.607	0.462
*Cs*. *annulata*	Correlation Coefficient	0.062	-0.090	0.056	0.086	0.170	-0.180	0.205	0.180	0.141	-0.139
	Sig.	0.744	0.636	0.769	0.651	0.370	0.342	0.277	0.341	0.459	0.464
*Cs*. *longiareolata*	Correlation Coefficient	-0.348	-0.138	-0.049	-0.103	0.106	-0.041	0.057	-0.013	0.199	0.171
	Sig.	0.060	0.468	0.796	0.588	0.577	0.831	0.764	0.945	0.291	0.365
*Cs*. *morsitans*	Correlation Coefficient	-0.294	0.161	0.107	0.011	-0.118	-0.097	0.000	-0.032	-0.228	0.182
	Sig.	0.115	0.395	0.572	0.955	0.534	0.611	1.000	0.866	0.225	0.335

## Discussion

Different factors influence the abundance and distribution of mosquito species including physicochemical factors, interspecific association, climate, vegetation, sources of nutrients and human activities [34]. The effects of physicochemical factors on the density of mosquito larvae were examined for the first time in Mazandaran Province, northern Iran.

*Culex pipiens* proved to be the dominant species in the province and its larvae were collected from all counties in the province, whereas *Cx*. *tritaeniorhynchus* and *Cx*. *torrentium* were not collected in a few localities. In a study by Nikookar *et al*., [35–37] across Mazandaran Province, *Culex pipiens* was the dominant species and Zaim [38] reported this species from 24 provinces of Iran. Compatibility of *Cx*. *pipiens* to different types of oviposition sites in the vicinity of human places with various degrees of physicochemical factors (especially degree of organic contamination, for example, ammonium ion) and suitable vegetation can probably be important in their interspecific interactions and justify its wide distribution in different counties of the province [16,39–41].

The most abundant anopheline species was *An*. *maculipennis* s.l., however, it was not collected from 16 villages in the province. A significant association between the abundance of *An*. *maculipennis* s.l. and all physicochemical factors was not observed in this study. It seems that changes in the agricultural patterns such as replacing rice cultivation with citrus gardens, reduced cattle husbandry (sources of blood for zoophilic behavior of adults of this species) are contributing factors to failure in collection of this species in some localities [42,43]. *Anopheles maculipennis* was reported to be the most common anopheline species in some provinces in the north and northwest of Iran [44–47].

Although no interspecific association was observed between *Culex* species, a number of such associations were observed between anophelines. The interspecific association coefficient for pairs of species *An*. *maculipennis* s.l/*An*. *pseudopictus* was the highest in Mazandaran Province followed by *An*. *maculipennis* s.l*/An*. *hyrcanus* and *An*. *pseudopictus/An*. *hyrcanus*. These species are “controphic species” and have common needs in a variety of habitats. It is affecting the biology, ecology and development of larvae because of competition for food, exposure to predators and susceptibility to pesticides. However, the relationship between controphic species (conspecific or heterospecific) that live together in a common habitat could be important in identifying vector habitats and also help the design and implementation of appropriate principles of vectors control programs [48].

In different studies in Iran, significant positive associations were detected (using coefficients other than C_8_) between the pairs of species *An*. *culicifacies/An*. *dthali* in Bashagard District, southern Iran [19], *An*.*dthali*/*An*.*turkhudi* in Isfahan Province [49] and *An*. *hyrcanus/An*. *pseudopictus* in Mazandaran Province, North of Iran [35]. The authors argued that these relationships are due to the same preferences in the choice of habitats and common food needs which are consistent with the results of the present study.

*Culex pipiens* showed a significant positive correlation with EC, alkalinity, total hardness and chloride. It is likely that changes in the cultivation of agricultural land for example converting them into citrus groves, as well as the use chemical fertilizers in the province, can alter the physicochemical characteristics of the water in the larval habitats, changes that may, in turn, alter the abundance of the species larvae [50]. These physicochemical parameters will be used as an energy source to increase the proliferation of algae and other micro-organisms (including bacteria) that serve as the main food for larvae [51], and provide chemical cues for choosing a suitable oviposition site by females and stimulate egg hatch [50]. It can also possibly lead to the interspecific interactions and mosquito community structure and its relationship to the risk of infectious diseases transmission in specific ecosystems [52].

As mentioned above, physicochemical factors are nutritional sources of water bodies. However, an excessive rise can have adverse effects on some aquatic organisms and subsequently reduce larval food sources [53]. For example, sulfate is a natural substance which includes sulfur and oxygen. It may be washed from the soil and added by other sources including decaying plant and animal matter, industrial and domestic sewages and farmland runoff in most water supplies [4]. A high level of sulphate showed significant influence on the abundance of *An*. *arabiensis* mosquito larvae [4].

A significant difference was observed between the density of *An*. *culicifacies* and calcium and EC; *An*. *turkhudi* and *An*. *superpictus* and total hardness in Sistan and Baluchestan Province of Iran. The authors believed that the larvae of *An*. *culicifacies* and *An*. *turkhudi* are more sensitive to physicochemical factors in different habitats compared with other species; this may explain the limited spread of the species in the country and the region [20] which is in agreement with our study.

In contrast with our research, no significant relationship was found between the abundance of the genera *Anopheles*, *Culex* and *Aedes* (formerly *Ochlerotatus*) with physicochemical and microbial parameters in Qom Province (central) and Bashagard district, southern Iran [19,21] and Egypt [7].

Even though other factors including temperature, pH, turbidity, nitrate, sulphate and phosphate from the mosquito larvae oviposition site did not show relationship with larval density after statistical analysis, their roles could not be disregarded. As is evident in others studies, there are a positive correlation between *Cx*. *pipiens*, *Cs*. *longiareolata*, *Cx*. *antennatus*, *Oc*. *caspius*, *Cx*. *vagans*, *Cx*. *decens*, *Cx*. *perexiguus*, *Cx*. *univittatus*, *An*. *multicolor* and temperature, pH and nitrate [7,8]; *An*. *arabiensis* and turbidity [17]; *An*. *sinensis* and sulphate [4]; *Culex quinquefasciatus* and phosphate [51].

A study on physicochemical characteristics of larval habitats and mosquito larval density in India revealed, although not significant, a positive correlation with pH and DO, whereas salinity, TDS and turbidity were negatively correlated with abundance of *Ae*. *albopictus*, *Cx*. *quinquefasciatus*, *Armigeres* s*ubalbatus*, *Ae*. *aegypti*, *Toxorhynchites sp* and *Lutziasp* larvae in containers, whilst significant negative correlation was noted between conductivity and larval density [54]. The investigators believed that these factors can be considered as predictor variables for density of mosquito larvae. This opinion is almost in agreement with our study that shows the importance of physicochemical factors in survival and population dynamics of mosquitoes, but this prediction value of physicochemical characteristics of larval habitats and mosquito larval density requires more investigation.

Although *Culex tritaeniorhynchus* was the second most abundant *Culex* species in the present study, the values of physicochemical factors were not helpful in explaining its abundances in our study. *Culex tritaeniorhynchus* prefers niches with wet muddy bottom emergent plant coverage and deeper water [24,55]. Therefore, this may indicate that other factors are involved in abundance of this species in the province.

As discussed, some of the data presented in others studies are in agreement [1,4,7,9] and some in contradiction [21] with the results of the present study which could be due to biological characteristics of different species that show different levels of tolerance to physicochemical factors.

As mentioned above, *Culex pipiens* is the most common specie across the province. It has generally been considered as an ornithophilic species and the most competent vector of WNV [56]. The species is opportunistic and bites both humans and animals; so, it can have the role of bridge vector between birds and humans and animals [57]. The wetlands are the prime locations for the emergence of the disease [58]. Detection of WNV in the mosquito populations and records of human infection with the virus in North and North West Iran [22,23] and availability of wetlands to migrating birds in Mazandaran Province may provide the ground for the entry and spread of the virus in the province. Biotic attributes such as the abundance and diversity of the host and abiotic conditions including physicochemical factors of the water are determinant factors in the epidemiology of West Nile fever [59].

Considering the impact of physicochemical factors on mosquito larval density, any changes in these factors without causing any negative effect on other forms of aquatic life, could possibly be considered as the basis for larval control strategies [17]. This also helps guide the public health efforts to prevent proliferation of the vectors of diseases including West Nile fever in the area. However, more investigation is needed on pathogens, predators, coverage of canopy, surface debris, algae and emergent plants in order to obtain solid evidence and get baseline information on mosquito larval habitat characteristics that may eventually be used for mosquito control programs. Environmental assessments should be considered before modifications such as alteration in cultivation, changes in the fertilizers used, environmental pollution and etc. are implemented in an area as they may change the abundance of the mosquito larvae.

### Conclusion

In conclusion, based on the findings of the present study, physicochemical factors of breeding sites including EC, alkalinity, total hardness and chloride may determine the distribution and abundance of *Cx*. *pipiens* in the area. Although seems important and expected to be observed, a significant positive correlation has not been detected for the rest of the species present in the study area. High interspecific association between pair of species *An*. *maculipennis* s.l/*An*. *pseudopictus* show that these species have common needs and adaptability for sympatry. These findings could be useful in comprehending the ecology of mosquito larvae that may be beneficial in designing and implementing larval control programs. This is the first study looking at the physicochemical parameters associated with larval abundance and diversity in Mazandaran Province. Further studies are needed to enable the use of this information in vector control programs with confidence.

### Limitations

It should be noted that there is a limitation to this study and that is we measured the physicochemical parameters only once in the whole of the study areas and sampling locations whereas the density of mosquito larvae measured every month. The possibility of relation of larval habitats physicochemical parameters and larval density fluctuation might have well been established should the trend of changes of the physicochemical parameters be determined, this has further research implications.
